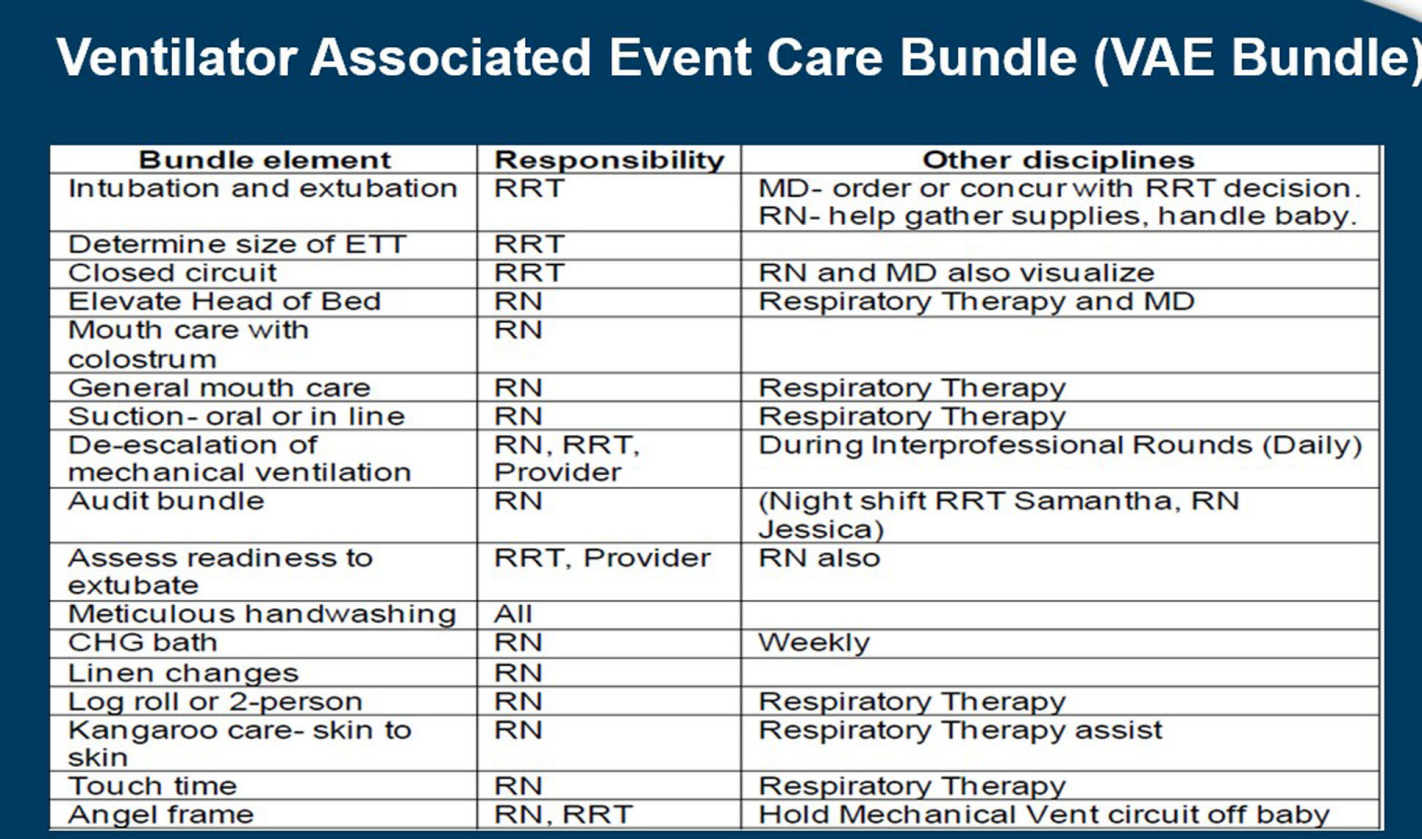# 360 Antimicrobial Resistance Surveillance in the Cuautla River, Mexico

**DOI:** 10.1017/ash.2026.10740

**Published:** 2026-06-23

**Authors:** Nicole Jackson

**Affiliations:** 1 MSOL, CIC, Atrium Health Navicent

## Abstract

**Background:** Ventilator-associated events (VAEs) are episodes of respiratory deterioration after stability during mechanical ventilation, posing significant risks for morbidity and mortality. Extremely low birth weight neonates (<750 g), particularly those born at 22–24 weeks gestation, are highly vulnerable. In our Neonatal Intensive Care Unit (NICU), eight VAEs occurred in the previous year, leading to prolonged ventilation, increased infection risk, and higher mortality. Reducing VAEs is essential for improving patient safety, health equity, and family experience while meeting NHSN benchmarks and regulatory goals. **Methods:** A structured, evidence-based approach was implemented using failure mode effects analysis (FMEA) to identify risk factors and guide interventions. A multidisciplinary team developed a formalized VAE prevention bundle, which included: - Formation of a multidisciplinary task force - Process measures tied to critical clinical tasks - Creation of an EHR tool for real-time compliance monitoring - Staff education and workflow alignment - Cultural engagement initiatives to reinforce best practices Pre- and post-intervention data were collected and analyzed for neonates <750 g per NHSN guidelines. **Result:** Post-implementation, VAE rates decreased from 9.8 to zero per 1,000 ventilator days, achieving an SIR ?1.0 and meeting national benchmarks. Mortality among extremely low birth weight neonates declined by 60%. Integration of EHR visualization and Power BI dashboards enabled real-time monitoring and sustained compliance. The initiative generated an estimated annual cost savings of $2.1 million through reduced length of stay and infection-related expenses. **Conclusion:** Structured care bundles, integrated data tools, and multidisciplinary engagement can eliminate VAEs in high-risk neonates. Broader adoption across NICUs could enhance patient safety, reduce healthcare costs, and improve outcomes systemwide. Innovative clinical practice plays a pivotal role in sustaining these improvements through adherence to prevention bundles, leveraging technology for real-time compliance, and fostering a culture of accountability. Standardized frameworks and education strategies can be scaled across institutions for continuous improvement. References Bondarev DJ, Ryan RM, Mukherjee D. (2024). The spectrum of pneumonia among intubated neonates in the neonatal intensive care unit. Journal of Perinatology, 44(9), 1235–1243. https://doi.org/10.1038/s41372-024-01973-9 Niedzwiecka T, Patton D, Walsh S, Moore Z, O'Connor T, Nugent L (2019, October). What are the effects of care bundles on the incidence of ventilator-associated pneumonia in pediatric and neonatal intensive care units? A systematic review. Journal for Specialists in Pediatric Nursing: JSPN. https://pubmed.ncbi.nlm.nih.gov/31332968/

**Figure:**
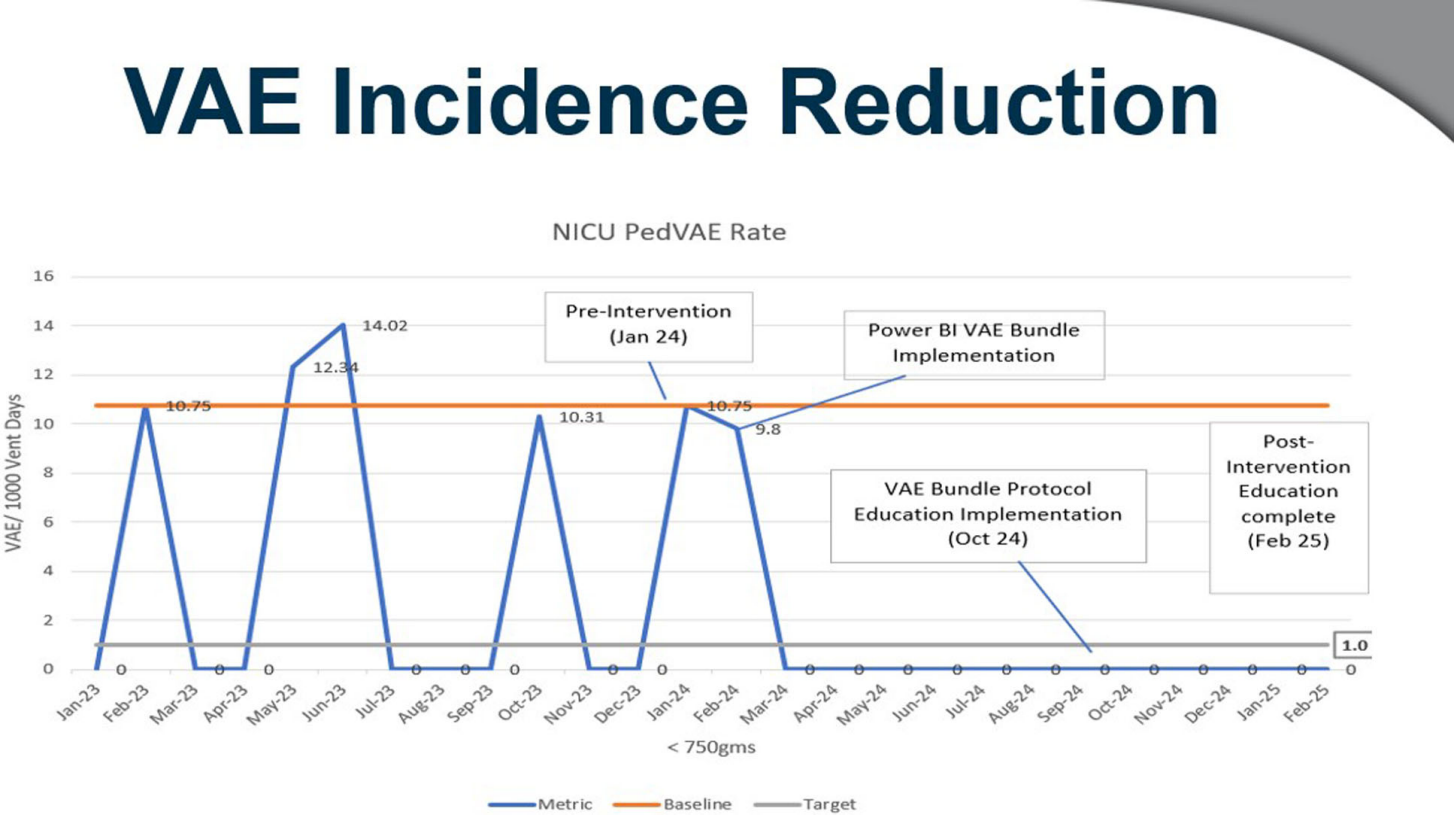

**Figure:**